# Assessing the reliability of tele-refraction for real time consultation with a remote optometrist

**DOI:** 10.1371/journal.pone.0299491

**Published:** 2024-06-24

**Authors:** Neha Kapur, Shalinder Sabherwal, Preeti Sharma, Javed Nayab, Patricia Koh Pei Chen, Soniya Srivastava, Atanu Majumdar

**Affiliations:** 1 Department of Ophthalmology, Dr Shroff’s Charity eye Hospital, Delhi, India; 2 Department of Public health, Dr Shroff’s Charity eye Hospital, Delhi, India; 3 Department of Optometry, Dr Shroff’s Charity eye Hospital, Delhi, India; 4 Base of Pyramid Innovation, EssilorLuxottica, Singapore; 5 Department of Statistics, Dr Shroff’s Charity eye Hospital, Delhi, India; University at Buffalo Jacobs School of Medicine and Biomedical Sciences: University at Buffalo School of Medicine and Biomedical Sciences, UNITED STATES

## Abstract

**Introduction:**

Uncorrected refractive errors pose a significant challenge globally, particularly in remote regions of low-middle income countries where access to optometric care is often limited. Telerefraction, which involves refraction by a trained technician followed by real-time consultation with remote optometrist, is a promising approach for such remote settings. This study aimed to evaluate the accuracy of this model.

**Methods:**

This prospective study, conducted in New Delhi, compared tele-refraction to in-person examinations. Trained technicians used a simple device, Click-check, to perform objective refraction and a tele-refraction platform to enter the findings of objective refraction. Final prescription was made after consulting a remote optometrist on that platform. Masked face-to-face optometrists served as the gold standard. The study involved refraction in 222 patients and 428 eyes.

**Results:**

Tele-refraction demonstrated a strong agreement with in-person optometry, achieving 84.6% in spherical correction and 81% conformity in spherical equivalent. The mean difference of spherical equivalent between the two arms was only 0.11 D. The consultation with a remote optometrist improved conformity of spherical equivalent by 14.8% over objective refraction. 82 percent eyes matched in best corrected visual acuity and 92 percent were within 0.1 logMAR difference. For cylindrical axis, 74% eye were within acceptable 10 degrees of difference. The mismatch amongst the individual trained technicians, in terms of difference between the tele-refraction arm and the face-to face optometrist arm was found to be significant for cylindrical axis and not for spherical power and spherical equivalent.

**Conclusion:**

Our study found tele-refraction by a trained technician comparable to refraction done by face-to-face optometrist. Tele-refraction, coupled with remote optometrist guidance can address the optometry resource gap in underserved areas. Thus, this model offers a transformative approach to enhancing the accessibility and quality of eye care services, which can significantly contribute to our efforts in achieving the global targets set by the World Health Organization for effective refractive error coverage. More standardized training for these technicians on ClickCheck^TM^ for detecting the cylindrical axis with better accuracy, can improve this model further.

## Introduction

Uncorrected refractive errors are a major cause of vision loss in India and globally [1]. The evaluation of refraction holds significant importance within the context of a comprehensive ocular assessment. Many individuals, particularly those residing in rural or remote locations in India, face challenges in accessing refractive services due to a shortage of trained optometrists in general and especially in these locations [2]. It is estimated that around 10 percent of adults in India have uncorrected refractive error and around 30 percent have uncorrected presbyopia [3].

Uncorrected Refractive Error can cause severe economic impact on family and the community [4]. Corrected vision allows the individual to return to a normal life of work and a traditional role in the family. But for years the formal practice of vision correction has been limited only in urban and peri-urban areas limiting the people living in remote villages to access it at an affordable price. In the Vision 2020: Right to Sight report [5], it is mentioned that the development of mid-level human resources for eyecare is essential for Southeast Asia. Globally, there is a shortage of specialized human resources such as optometrists, to provide quality refraction.

The rapid evolution of telecommunication and digital technologies has revolutionized the healthcare landscape, introducing innovative solutions to improve access to medical services, especially in underserved and remote areas. Telemedicine, the remote provision of healthcare services, has emerged as a transformative approach to bridge the geographical and logistical gaps between patients and healthcare providers [6–8].

Within the realm of telemedicine, telerefraction has emerged as a compelling tool, specifically addressing the needs of optometry by enabling real-time consultations with remote optometrists worldwide [9].

Telerefraction, also known as remote or tele-optometry, offers a potential solution to this challenge [9]. This innovative approach leverages advancements in digital technologies and telecommunication to enable remote optometric assessments. Through telerefraction, individuals can receive accurate measurements of their refractive errors, visual acuity, and other basic optometric evaluations without the need to travel long distances to a physical eye care facility.

OneSight Essilor Luxottica Foundation’s tele-refraction model consists of an on-demand service platform to connect a primary vision care provider, trained technician trained by Essilor [10,12] and their customer with a qualified optometrist who remotely oversees the refraction process in real-time via video consultation and can update the refraction measurements on the platform and sign that off. Trained technicians in this model first conduct history taking and unaided visual acuity of their customer. They then use a device called ClickCheck^TM^ [13], to obtain the starting point refractive power. ClickCheck^TM^ is a portable device manufactured by Essilor International that aids in estimating refractive error through adjusting the dial on a plastic tube. It is based on the Badal optometer principle [14]. Here the image size is kept constant while the target distance and stimulus to accommodation is varied using an auxillary lens.

The trained technician, then, connects to an optometrist remotely through the telerefraction application. The remote optometrist will receive the data of the customer in his/her application and start guiding the trained technician to perform subjective refraction. The remote optometrist is able to see the customer and provide real-time consultation as well. Those in need of further testing are referred to a secondary eyecare facility.

D A Goss et al did a review of the literature on the reliability of refraction where they found the intra-examiner reliability and inter-examiner reliability of subjective refraction in most studies were close to 80 percent agreement within +/- 0.25D and 95 percent agreement within +/- 0.50D for spherical equivalent, sphere power, and cylinder power[15]. JJ Waline et al in their study showed that 80% of the eyes undergoing 2 measurements of retinoscopy by the same examiner had a shift of axis >/ = 5degree and 40% had a shift of >/ = 10degrees [16]. No studies have been reported till now to assess the accuracy and real-time tele-refraction with a remote optometrist.

TeleRefraction has been limited in availability and there have been very few studies comparing tele-refraction with face-to-face refraction [9–11]. So, the purpose of this study is to assess the accuracy and reliability of real-time tele-refraction by a trained technician with a remote optometrist versus face-to-face (f2f) examination by optometrist to diagnose and manage refractive error. If found accurate, this model can improve access to quality refractive services to people in remote locations.

## Methods

This is a non-inferiority diagnostic accuracy design/prospective study conducted at Dr. Shroff’s Charity Eye Hospital, Daryaganj, New Delhi. The study was approved by the Institutional Review Board of Dr. Shroff’s Charity Eye Hospital(IRB/2023/Jan/136) and was performed in accordance with the tenets of the Declaration of Helsinki. A verbal consent was obtained from participants to conduct refraction at two stations and an informed written consent for using anonymized data for research, after explaining the details of the study to the participants. The recruitment period was from 15th March 2023 to 19th April 2023.

### Patients

All types of patients reported to the base hospital in the age group 18 to 55 years, with refraction values within ± 6 diopters spherical and ± 2 diopters of the cylinder were included in the study. Those with any corneal, retinal or any obvious ocular pathology were excluded by a clinical optometrist, before sending them to be included in the study. Patients having a dull glow (reflex) as in high myopia, vitreous haemorrhage or cataract were also excluded.

### Trained technicians

The technicians were trained on visual acuity measurement, ClickCheck^TM^, trial lens usage and reading of a prescription, with an experience of at least 20 recorded refractions on the TeleRefraction platform. Those not fluent in Hindi (local language) and those not willing to travel to Delhi for this study were excluded.

### Optometrist for face-to-face examination

The optometrists for f2f examination and remote optometrists had a minimum of 2 years of clinical experience. A test (prior to start of the study) on 20 subjects were done to ensure the standards across them are similar. Those who were not fluent in Hindi were excluded.

Selected participants underwent a history-taking procedure by trained technicians. The uncorrected distance visual acuity (UDVA), pinhole vision, and best-corrected distance visual acuity (BCVA) were recorded.

Objective refraction (spherical and minus cylindrical powers and axis) was carried out using ClickCheck^TM^ device. The data was entered on the Telerefraction application and then the trained technician got connected to an optometrist remotely. The remote optometrist received the data of the subject in his/her application. The Technician followed the instruction from the optometrist to complete the subjective refraction.

The Optometrist guided the trained technician on what trial lenses to be placed on the trial frame for the subject as starting point. The Optometrist could control the digital visual acuity to be seen by the subject while the trained technician changes the trial lenses. The optometrist then updates the refraction measurements in the application as the trained technician adjusts the trial lens power until the best vision is achieved. Upon completion of the refraction process, the Optometrist enters the final prescription on the application with the BCVA. The Optometrist signs off on the prescription for the technician to prepare glasses for the subject. Should there be a need for further testing, the Optometrist can refer the patient to the nearest eye hospital.

The trained technicians can also choose to connect only to Optometrists who are able to speak the local languages. In case of issues such as call drops during a consultation, the trained technician also has the option of reconnecting with the same Optometrist to continue the consultation.

Subsequently, each patient went through f2f refraction (gold standard) with another optometrist who performed objective refraction using Heine^R^ BETA 200 streak retinoscope and the final prescription was released after performing subjective refraction on each subject. The face-to-face optometrist was masked to the finding of tele-refraction.

### Sample size

The study aimed to compare the difference between two variables (refractive errors of a person once assessed through tele-refraction, and, next, assessed face-to-face by a trained optometrist), and a difference of greater than ±0.5 D was considered unacceptable. The sample size was calculated to answer in what proportion of cases trained technicians report the refraction accurately (the difference lies within ± 0.5D). The sample size required to estimate that proportion within ±10%, with a 95% CI was 200. Eyes. We assumed maximum variance and a design effect of 2.

### Statistical analysis

The study involves estimating the rates of conformity in terms of spherical and cylindrical dioptres, axis and visual acuity. The rates of conformity have been expressed as percentages and we have reported the estimates with their 95% confidence interval. The study also compares the mean of those parameters between the two main groups and across subsamples classified by the level of conformity for which we have applied t-test or ANOVA depending on the situation.

## Results

**Fig 1:**
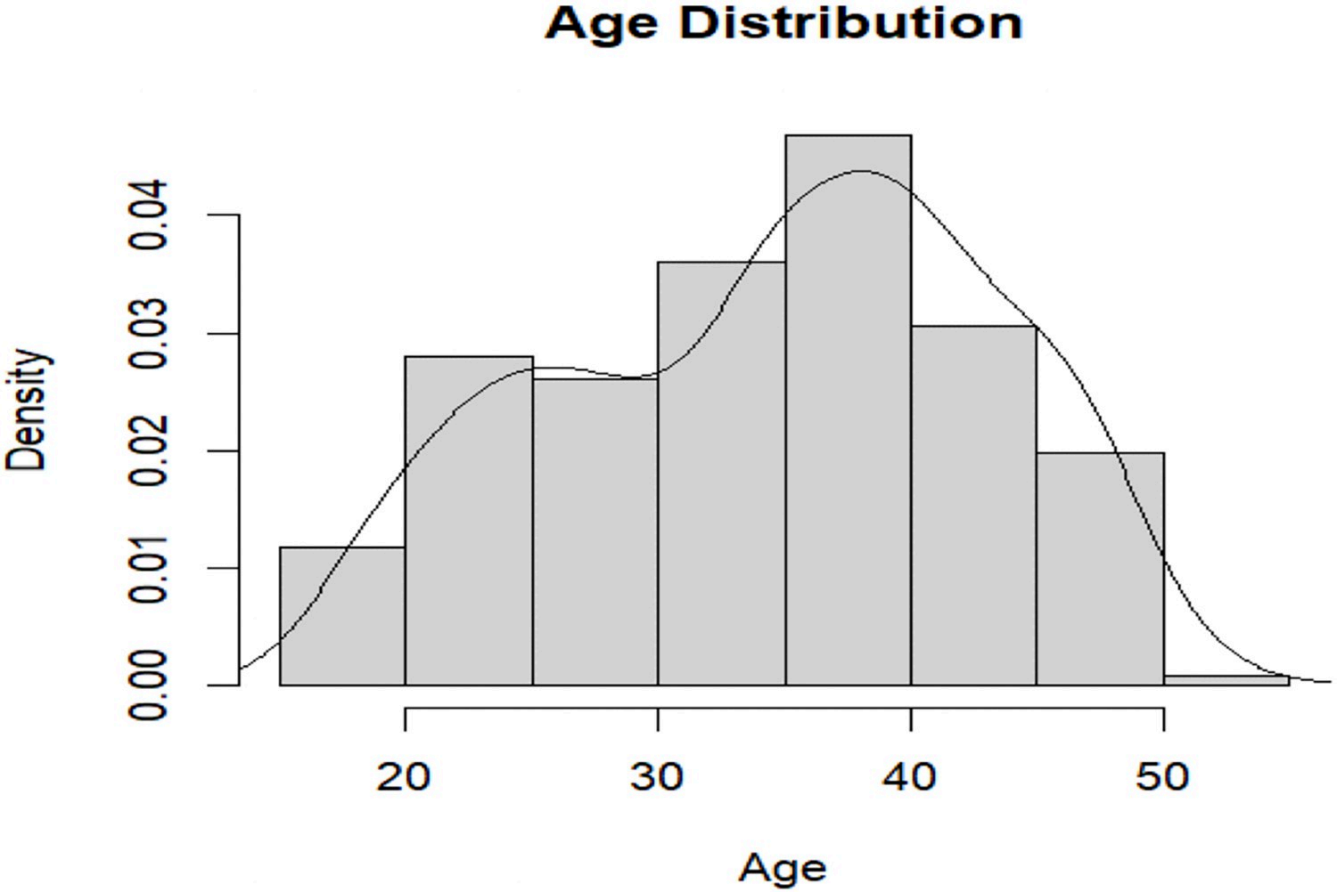
Age-distribution of patients.

A total of 222 patients were enrolled between the period of March 15, 2023, and April 11, 2023. The maximum patients were in the age-group of 35–40 years as shown in Fig 1.

### Conformity in terms of spherical dioptres, cylindrical dioptres and axis between the eye mitras and f2f optometrists

**Fig 2:**
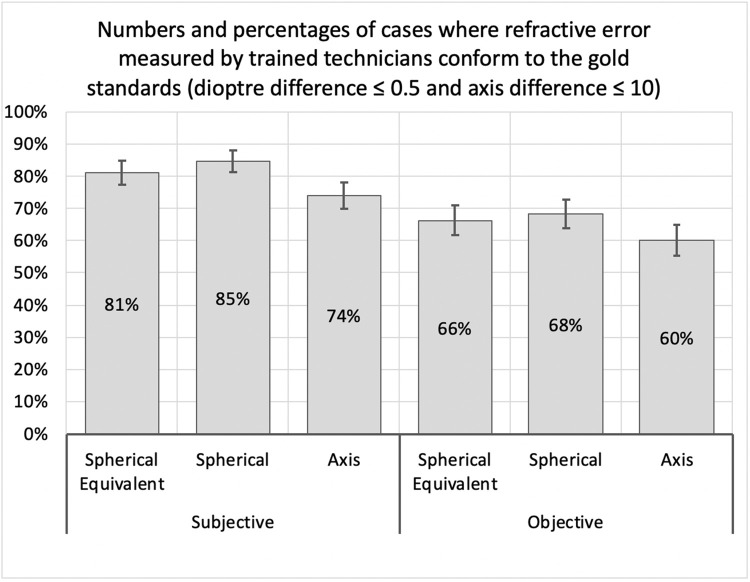
Conformity in terms of spherical equivalent, spherical power and axis.

Two f2f optometrists and one remote optometrist and eleven trained technicians participated in the study. Of the 428 eyes refracted by trained technicians, for which data were available, 81 percent were within the acceptable range for spherical equivalent (within 0.5D of face-to-face optometrist), 84.6 percent were within the acceptable range for spherical correction (within 0.5D of face-to-face optometrist) and 74 percent withing the acceptable range for cylindrical axis (within 10 degrees) after the final input from the remote optometrist (Fig 2).

The spherical equivalent power obtained from only objective measurement was 66% conforming to the gold standards. By using tele-consultation, connecting with the remote optometrist, 14.8 percent additional eyes conformed to the accepted range (over and above those with objective refraction) (Table 1).

**Table 1:** Numbers and percentages of cases where refractive error measured by trained technicians conform to f2f optometrist.

		N	Number of eyes with refraction within the accepted difference from that of face-to-face optometrist	Percent-age	95% CI		Increase in agreement due to tele-refraction input
**Objective** **Refraction**	Spherical Equivalent	421	279	66.27%	61.8%	70.8%	NA
	Spherical	421	287	68.17%	63.7%	72.6%	NA
	Axis	418	251	60.05%	55.4%	64.7%	NA
**Subjective** **Refraction**	Spherical Equivalent	428	347	81.07%	77.4%	84.8%	14.8%
	Spherical Power	428	362	84.58%	81.2%	88.0%	16.4%
	Axis	427	316	74.00%	69.8%	78.2%	14.0%

### Difference in the values of spherical dioptres, cylindrical dioptres and axis as measured by trained technicians and f2f optometrists

The difference between mean spherical power for tele-refraction arm (trained technician and remote optometrist) and f2f optometrist was 0.01, that for cylindrical power was 0.21 and that for final spherical equivalent was 0.11 after the final subjective refraction (final prescription). The difference between the mean cylindrical axis between the two arms was 6.3 degrees (Table 2).

**Table 2:** Mean difference in diopters and axis between refractive error measured by face-to-face (f2f) optometrist and the tele-refraction arm (trained technician with remote optometrist).

Final Prescription	N	Optom f2f		TeleRefraction (Technician + remote Optometrist)		Paired Diff =		p-Value (t-test)
						Optom f2f - TeleRefraction		
		Mean	SD	Mean	SD	Mean	SD	
Spherical	428	-0.11	1.40	-0.10	1.34	-0.01	0.76	0.748
Cylinder (subjective)	428	-0.39	0.61	-0.19	0.41	-0.21	0.48	*0*.*000*
Axis (subjective)	427	27.04	46.46	20.71	42.88	6.32	47.69	*0*.*006*
Spherical Equivalent (subjective)	428	-0.31	1.44	-0.20	1.35	-0.11	0.75	*0*.*002*

### Conformity in terms of visual acuity

**Fig 3:**
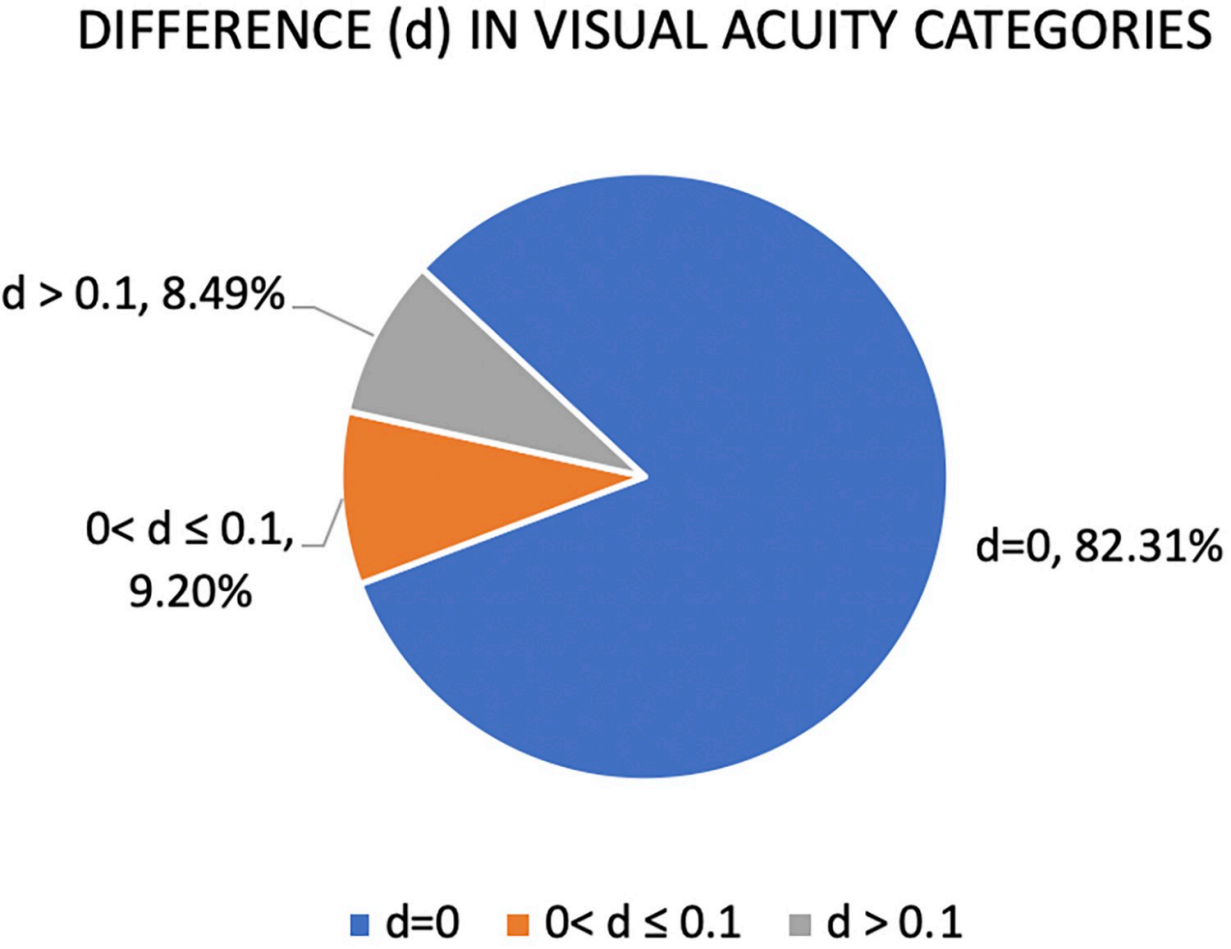
Proportion of eyes categorized according to difference in best corrected visual acuity.

82 percent of the patients matched in terms of final corrected visual acuity between the two arms and around 92 percent were within 1 line of difference on logMAR chart (0.1 logMAR difference). For those with difference up to one line, more than 84 percent conformed to the acceptable difference in spherical equivalent (Fig 3).

### Difference in the spherical power and axis as assessed by the two arms of the study in subsamples classified by the level of conformity in terms of visual acuity

Eyes with difference in BCVA of 0 line had a mean spherical equivalent difference of 0.11 D and a mean axis difference of 4.3 degrees. When the line difference became more than 0.1logMAR, the mean spherical equivalent difference was 0.26D whereas the mean axis difference increased to 16.8 degrees (Table 3). The difference of mean spherical power, spherical equivalent, or cylindrical axis, between those with up to one line of difference and more than one line of difference in BCVA was not found to be significant.

**Table 3:** Difference in mean spherical equivalent, spherical correction and cylindrical axis related to the difference in BCVA.

Difference (d) in Visual Acuity Category in logMAR	Difference in mean		
	Sph Eq	Sph power	Axis
d = 0	-0.11 ± 0.6	-0.03 ± 0.56	4.32 ± 44.19
0 ≤ d ≤ 0.1	-0.13 ± 0.6	-0.04 ± 0.57	5 ± 46.69
d > 0.1	-0.26 ± 1.19	-0.06 ± 1.28	16.86 ± 52.69
p-value or difference up to 0.1 and more than 0.1logMAR	0.518	0.937	0.206

BCVA- Best corrected visual acuity.

### Conformity in terms of spherical dioptres and axis as assessed by the trained technicians and f2f optometrists in subsamples classified by the level of conformity in terms of visual acuity

For the patients where the BCVA difference was up to one line of logMAR chart, 84 percent of prescriptions were within the cut-off level of 0.5 D for spherical equivalent and 87 percent were within the cut-off level of 0.5 D for spherical correction. This reduced to 60 percent and 69 percent respectively for those with more than one line of difference in BCVA. The difference was found to be statistically significant. For the cylindrical axis, the conformity rates decreased from 75 percent to 63 percent, and this was not found to be statistically significant (Table 4).

**Table 4:** Difference in conformity rates related to the difference in BCVA.

Difference (d) in Visual Acuity Category	Conformity rates (%)		
	SphEq	Sph Dioptre	Axis
d = 0	86.4%	90.2%	78.0%
0 ≤ d ≤ 0.1	84.2%	86.9%	75.3%
d > 0.1	60.0%	68.6%	62.9%
p-value (between those with difference of up to 0.1 and more than 0.1)	0.001	0.008	0.161

BCVA- Best corrected visual acuity.

### Check for the random effect of the trained technicians in the assessment of BCVA, spherical dioptres and axis

We tested if the difference in BCVA, spherical equivalent and spherical power between the f2f optometrist arm and tele-refraction arm varied across different trained technicians. Only the difference in the cylindrical axis was found to vary significantly across different trained technicians (Table 5).

**Table 5:** Variation in difference (mean ± SD) in measurement across different trained technicians.

Trained Technician	N	Difference in BCVA	Difference in in Spherical Equivalent	Difference in Spherical Correction	Difference in Cylindrical Axis
1	48	-0.02 ± 0.27	-0.15 ± 0.51	-0.08 ± 0.48	-5.42 ± 32.45
2	30	-0.02 ± 0.14	-0.14 ± 0.99	-0.13 ± 1.18	11.73 ± 59.6
3	34	-0.01 ± 0.09	-0.25 ± 0.9	-0.1 ± 0.91	7.35 ± 52.92
4	26	0.03 ± 0.1	-0.05 ± 0.52	0.1 ± 0.45	13.46 ± 49.79
5	26	-0.01 ± 0.06	-0.12 ± 0.48	-0.01 ± 0.36	-5.93 ± 54.6
6	34	0.03 ± 0.08	-0.17 ± 0.43	-0.01 ± 0.38	28.82 ± 43.19
7	38	0.02 ± 0.06	-0.08 ± 0.45	-0.1 ± 0.43	-24.12 ± 51.23
8	38	-0.01 ± 0.05	-0.26 ± 1	-0.06 ± 0.92	19.47 ± 52.24
9	38	0 ± 0.02	-0.18 ± 0.58	-0.05 ± 0.5	10.92 ± 47.06
10	48	0.01 ± 0.05	0.03 ± 1.13	0.13 ± 1.11	1.54 ± 29.51
11	64	0.02 ± 0.05	-0.02 ± 0.71	0.06 ± 0.82	11.71 ± 46.76
p-Value		0.447	0.775	0.891	*0*.*000 *

## Discussion

It would usually take at least 2 to 3 years to train an optometrist and at least 3 to 5 years to train an ophthalmologist. In addition to the high cost and long training duration, most people would operate in the urban or peri-urban community. In areas where such specialized human resources are scarce, technicians can be trained on basic refraction process in a shorter timeframe and be connected to a qualified optometrist. Telerefraction application allows quality prescription with inputs from a remote optometrist.

As per our results, the tele-refraction model was found to be an acceptable model with more than 82 percent of the eyes having the same BCVA and more than 91 percent being within one line of BCVA difference as compared to results from face-to-face refraction. Around 84 percent of the eyes were found to be within the acceptable range of spherical equivalent. In eyes with no difference in BCVA (in 82% of the eyes), a clinically insignificant difference was found between mean spherical equivalent of final prescription obtained from tele-refraction and face to face refraction (-0.11D). When the difference in BCVA between the two arms increased from 0 line to 1 line, the difference in spherical equivalent increased from 0.10D to 0.26 D only, whilst the mean cylindrical axis difference increased from 4 degrees to 12 degrees.

The mean difference in the cylinder axis between the 2 arms was 6.32 degrees, this is less than the acceptable range of 10 degrees. Hence, resulting in statistically and clinically insignificant difference. As the difference in BCVA between the two arms increased to 1 line or more, the difference in mean spherical equivalent increased from 0.13 to 0.26 diopters only while the difference in mean cylindrical axis increased from 4 to 17 degrees. Moreover, the mismatch amongst the individual trained technicians, in terms of difference between the tele-refraction arm and the face-to face optometrist arm was found to be significant for cylindrical axis and not for spherical power and spherical equivalent. Thus, there seems to be a scope to improve the detection of cylindrical axis by these trained technicians using ClickCheck^TM^. Even in a validation study for ClickCheck^TM^, it was recommended that the device could be enhanced on its cylindrical power and axis estimation [13].

Another observation was that during the refinement of prescription through tele refraction, the remote optometrist could refine the prescription of axis of the cylinder, spherical equivalent and spherical correction by 14 to 16 percent, leading to an improvement in conformity with the face-to face optometrist model. This shows that TeleRefraction using a remote optometrist can improve the quality of prescriptions. In previous studies comparing another technical device, Netra, being used by technicians without supervision, results were found to be inferior to standard refraction by an optometrist [17].

The strength of our study is that the face-to-face optometrist and the trained technicians were masked to the findings of each other. The groups to which the data belonged was also not disclosed to the statistician.

One of the limitations of our study is that cases with any obvious ocular pathology, poor glow or high powers were excluded, thus the results of our study may not be valid for such patients. Such patients may need referral for refraction by a trained optometrist. Also, in the TeleRefraction arm, inaccuracy was more in the cylindrical axis with less refinement possible. At 10 degrees cylinder as a limit, the agreement with gold standard is expected to be more than 90 percent but was found to be less in our study.

Also, the mismatch varied across different trained technicians. We recommend that more standardized training is required for these technicians on ClickCheck^TM^ [13] for detecting the cylindrical axis with more accuracy.

## Conclusion

The World Health Organization has endorsed effective refractive error coverage as one of the important indicators for universal health coverage and global targets have been set for this indicator [18]. TeleRefraction provides a suitable solution to the human resource crisis in optometry by providing access to qualified professionals to trained technicians through telemedicine. This has a potential to increase coverage and the quality of refractive services in underserved locations and thereby have an impact on effective coverage. Appropriate training of technicians and infrastructure are integral to any tele-refraction approach.

## Supporting information

S1 Checklist (DOCX)

## Abbreviations

UDVA: uncorrected distance visual acuity
BCVA: best-corrected distance visual acuity
f2f: face to face refraction
